# Citrus Tristeza Virus Genotype Detection Using High-Throughput Sequencing

**DOI:** 10.3390/v13020168

**Published:** 2021-01-23

**Authors:** Rachelle Bester, Glynnis Cook, Hans J. Maree

**Affiliations:** 1 Department of Genetics, Stellenbosch University, Private Bag X1, Matieland 7602, South Africa; rachelle@sun.ac.za; 2 Citrus Research International, P.O. Box 28, Nelspruit 1200, South Africa; glynnis@cri.co.za; 3 Citrus Research International, Stellenbosch, P.O. Box 2201, Matieland 7602, South Africa

**Keywords:** CTV, HTS, next-generation sequencing, variants

## Abstract

The application of high-throughput sequencing (HTS) has successfully been used for virus discovery to resolve disease etiology in many agricultural crops. The greatest advantage of HTS is that it can provide a complete viral status of a plant, including information on mixed infections of viral species or virus variants. This provides insight into the virus population structure, ecology, or evolution and can be used to differentiate among virus variants that may contribute differently toward disease etiology. In this study, the use of HTS for citrus tristeza virus (CTV) genotype detection was evaluated. A bioinformatic pipeline for CTV genotype detection was constructed and evaluated using simulated and real data sets to determine the parameters to discriminate between false positive read mappings and true genotype-specific genome coverage. A 50% genome coverage cut-off was identified for non-target read mappings. HTS with the associated bioinformatic pipeline was validated and proposed as a CTV genotyping assay.

## 1. Introduction

High-throughput sequencing (HTS) is a powerful tool with a range of applications. In the plant physiology field, HTS has played a significant role in genome sequencing [1,2,3,4,5,6,7], plant transcriptomics [8,9,10,11,12], plant–pathogen interaction studies [13,14,15,16], and also in the discovery of novel viruses and virus variants [17,18,19,20,21,22,23,24,25,26,27,28]. This technology can provide the complete virome of a plant in a single assay with a high degree of specificity that enables the identification of mixed variant infections of a single virus species. This information can be utilized to study virus population structure, ecology, or evolution or can be used to differentiate between virus variants that may contribute differently toward disease etiology [28].

*Citrus tristeza virus* (CTV) is a single-stranded, positive-sense RNA virus of approximately 19.3 kb in the genus *Closterovirus*, family *Closteroviridae* [29,30,31]. Previous studies showed a high degree of sequence divergence with at least eight phylogenetic separate genotypes identified [32,33,34]. The term genotype is used to group genetically similar genomes together [32,33]. These genotypes are found worldwide as members of mixed populations within a single host plant; therefore, complicating the genotypic and phenotypic associations since different combinations of genotypes affect symptom expression and disease severity [31,34,35]. Depending on the genotype and the citrus scion–rootstock combination, CTV can cause three syndromes named tristeza (quick decline), stem pitting, and seedling yellows [35]. CTV has caused major tree losses resulting in a severe decline in production [35]. Both quick decline and stem pitting can result in tree loss. The determinants of stem pitting development in the CTV genome are not yet known, but the expression of genes p33, p13, and p18 appear to play a role [36,37]. It has been shown that certain deletion combinations of these three genes can increase stem pitting symptoms, while other combinations resulted in reduced stem pitting [37]. A previous study using CTV infectious clones also showed that expression of these genes is needed for the systemic infection of the full host range of CTV, but different genes were specific for different hosts [38]. CTV transmission through the brown citrus aphid, has also been shown to be influenced by different CTV genotypes and that by creating genotype recombinants the transmission rate could be altered [39]. These studies suggest complex interactions between the genotypes and the citrus host and potentially the susceptibility of the tree to CTV.

Some citrus-producing countries have reduced the negative effects of CTV by applying a cross-protection management strategy. Plant material is inoculated with mild-strain sources of CTV to decrease the damaging effects of secondary infections that can be introduced by aphid vectors. Mild-strain cross-protection is applied in Australia [40], Brazil [41], Peru [42], and also South Africa, where the strategy has significantly extended the productive life of grapefruit varieties [43]. It is not known which genotypes are essential for cross-protection due to the complexity of mixed populations and genome diversity. In South Africa, the cross-protecting sources were empirically selected and comprised mixed-genotype infections that prevent attributing cross-protection to specific genotypes [44]. Therefore, it is important to resolve which genotypes contribute to cross-protection in a specific citrus host. The characterization of sources and the identification of single-variant CTV sources are essential to elucidate cross-protection and contribute to CTV diversity.

To enable population studies and potentially to resolve the cross-protection phenomenon, genotype-specific assays are required. A number of attempts have been made, including molecular markers and reverse transcription polymerase chain reaction (RT-PCR) [33,45,46,47]. However, no single CTV genome region is informative enough to differentiate all genotypes, and different target regions were selected for the known genotypes. The second limitation of genotype-specific RT-PCR assays is that they have to be re-evaluated as new genotypes are identified. The determination of full genomes and the utilization of high-throughput sequencing can potentially circumvent these limitations if a bioinformatic pipeline can be established for genotype identification. As the CTV genotype boundaries are becoming more defined or more CTV genotypes are identified, it will be possible to re-analyze previously generated data sets to refine CTV genotype differentiation in those samples without a second round of sampling, RNA extraction, and HTS.

In this study, an HTS bioinformatic pipeline was evaluated for the identification of CTV single or mixed genotype infections. The influence of data size on genotype-specific read mapping using simulated and real data sets were calculated to determine parameters to discriminate between false positive read mappings and true genotype-specific genome coverage. The study highlighted the importance of accurate reference databases and showed that it is possible to detect known and unknown CTV genotypes using an HTS assay.

## 2. Materials and Methods

### 2.1. Identifying Reference Sequences

All the CTV complete genome sequences (82) were extracted from the National Center for Biotechnology Information (NCBI) GenBank and aligned using the Multiple Alignment Fast Fourier Transform (MAFFT) version 7 [48]. An unrooted phylogenetic network was constructed using SplitsTree4 4.16.2 [49] to identify the different genotype groups. A representative genome for each CTV genotype was selected from NCBI GenBank (AF260651, NC_001661, EU937519, KU589212, KU883267, KU883265, MH051719, MK033511, JQ798289, MH323442, MH323441), and a multiple alignment was constructed using MAFFT. Pairwise comparisons between the representative sequences were performed with the CLC Genomics Workbench 11.0.1 (CGW) (Qiagen), and similarity plots were constructed using SimPlot 3.5.1 [50] with each of the genotype sequences as a query.

### 2.2. Simulated Data Generation

Synthetic HTS reads were simulated using ART-MountRainier version 2.5.8 [51]. A representative genome for each CTV genotype was selected from the NCBI GenBank (AF260651, NC_001661, EU937519, KU589212, KU883267, KU883265, MH051719, MK033511, JQ798289, MH323442, MH323441) and used to generated reads separately per reference. The HiSeq 2000 (100 bp) sequencing system was used for 100 nt paired-end read simulation. Different read coverage quantities were simulated by creating seven data sets of different numbers of read pairs per reference sequence (1000, 5000, 10,000, 50,000, 100,000, 500,000, 1,000,000) with a mean read pair distance of 300 and standard deviation of 50.

Single variant infections were evaluated by constructing 77 different single genotype data sets (11 genotypes of seven different data set sizes). To evaluate mixed variant infections, genotype-specific data sets were combined in different combinations of varying concentrations. The first combination of data sets included all the genotypes per data set size (e.g., 1000 reads pairs per genotype, 2000 read pairs per genotype, etc.) (7 combinations). The second combination of data sets included all genotype-specific data except for one genotype per data set size (e.g., 1000 read pairs each for ten of the eleven genotypes to create eleven different data sets) (77 combinations). The third grouping of data sets included all the combinations where the same amount of data for each genotype was added except for one genotype that was varied for all the data set sizes (e.g., 1000 read pairs per genotype except for one genotype that varied from 5000 to 1,000,000 to create 66 different data sets) (462 combinations). The fourth grouping of data sets included the 0, 1000, and 1,000,000 data set sizes of the different genotypes to create all possible genotype combinations of these three data set sizes (177,147 combinations).

To evaluate the influence of host genome reads on the CTV genotype read mappings, 40 million paired-end reads were simulated using ART-MountRainier from the *Citrus sinensis* host genome (C.sinensis_Hzau_v2.0_genome) [4]. Host reads were mapped randomly to the 11 CTV reference genotypes using CGW (default read mapping and 95% similarity over 90% read fraction read mapping).

### 2.3. Read Mapping

The different data sets created representing different genotype combinations in varying concentrations were mapped concurrently, unless otherwise stated, to the eleven representative genotype sequences using the Burrows–Wheeler Alignment Tool (BWA) version 0.7.13 [52] (Supplementary File S1). The resulting sam files were filtered to retain only the mapped reads that had a 95% similarity for 90% of the read fraction (Supplementary File S1). The filtered sam files were sorted, and the read depth at each position was computed using samtools version 1.10 [53] (Supplementary File S1). The genome coverage (span) for each reference genome was calculated by counting all the non-zero read depth positions (Supplementary File S1).

The 1000 and 1,000,000 data subsets per genotype were also individually mapped to each of the representative genotypes separately using the abovementioned pipeline.

### 2.4. Data Visualisation

The combined read mapping results consisting of the number of reads and coverage percentage per reference sequence for each of the combinations were visualized using a custom-made Shiny application dependent on the R statistical computing environment (R Core Team, http://www.R-project.org/). The results from each of the abovementioned combinations can be visualized at https://rbester.shinyapps.io/CTV_mapping_11Dec/ or in Supplementary File S2.

### 2.5. Citrus HTS Data Generation

Three citrus samples infected with different CTV genotypes and one healthy citrus sample were selected for HTS. Total RNA was extracted from one gram of leaf midribs of each sample using a modified Cetyltrimethylammonium Bromide (CTAB) extraction protocol [54].

Two-step RT-PCRs were performed to determine the CTV genotype status of each plant. Complementary DNA (cDNA) was synthesized from 1 μg of total RNA using 0.15 μg of random hexamers (Promega) and Maxima reverse transcriptase (Thermo Scientific, Waltham, MA, USA) in a final volume of 20 μL according to the manufacturer’s instructions. A 2-μL aliquot of cDNA was added to 25 μL of PCR reaction mixture containing 1 × KAPA Taq buffer A (Roche, Basel, Switzerland), 0.2 mM dNTP mix (Thermo Scientific, Waltham, MA, USA), 0.4 μM forward and reverse primers (IDT, Coralville, IA, USA) (Table 1), and 1.25 U/μL KAPA Taq DNA polymerase (Roche, Basel, Switzerland). Cycle conditions for the different assays included an initial denaturation step at 94 °C for 5 min, followed by 35 cycles of 94 °C for 30 s, primer specific annealing temperature (Table 1) for 30 s, and elongation at 72 °C for 30 s, and a final extension of 72 °C for 7 min.

Four Ribo-depleted RNA libraries, one from each citrus plant, were constructed with the Illumina TruSeq Stranded Total RNA Sample Preparation kit with Plant Ribo-Zero at Macrogen (Seoul, Korea). Paired-end HTS (2 × 100 bp) was performed on an Illumina NovaSeq 6000 instrument (Macrogen, Seoul, Korea).

Adapter sequences were removed from the Illumina data, and data were trimmed for quality using Trimmomatic [55] (SLIDINGWINDOW of 3 nts with Q20, MINLEN of 20 nts).

The quality trimmed data were subjected to de novo assemblies using the St. Petersburg genome assembler (SPAdes) 3.14 [56] and CGW (default parameters). The de novo assembled scaffolds and contigs were identified using BLAST+ standalone against a local copy of the NCBI GenBank nucleotide database using the Blastn algorithm.

All the quality trimmed reads were mapped to the reference genomes, and the genome coverage (span) calculated using the abovementioned pipeline (Supplementary File S1).

A sample specific consensus sequence was generated for each genotype present in the sample using CGW (read mapping: 95% similarity; 90% read fraction, consensus: conflict resolution = vote, low coverage = fill from reference). The reference sequences used for the concurrent read mappings were replaced by the sample-specific consensus sequences for the T30, S1, HA16-5, T68, and A18 genotypes in the reference list (Supplementary Files S3 and S4), and read mapping was performed using the BWA pipeline.

## 3. Results

### 3.1. CTV Genotype Selection

**Figure 1:**
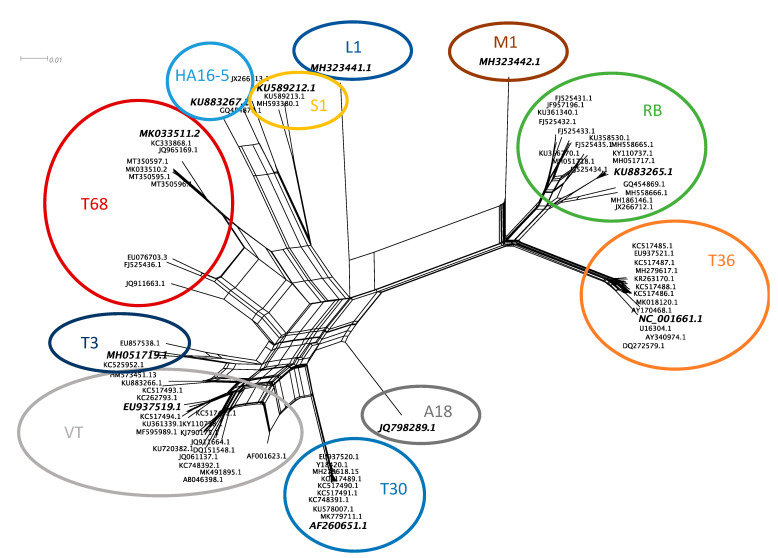
Neighbor network reconstruction of the complete genomes of citrus tristeza virus (CTV). Genotype groups are indicated with colored circles, and a representative accession from each group is highlighted with increased font size and italics.

**Figure 2:**
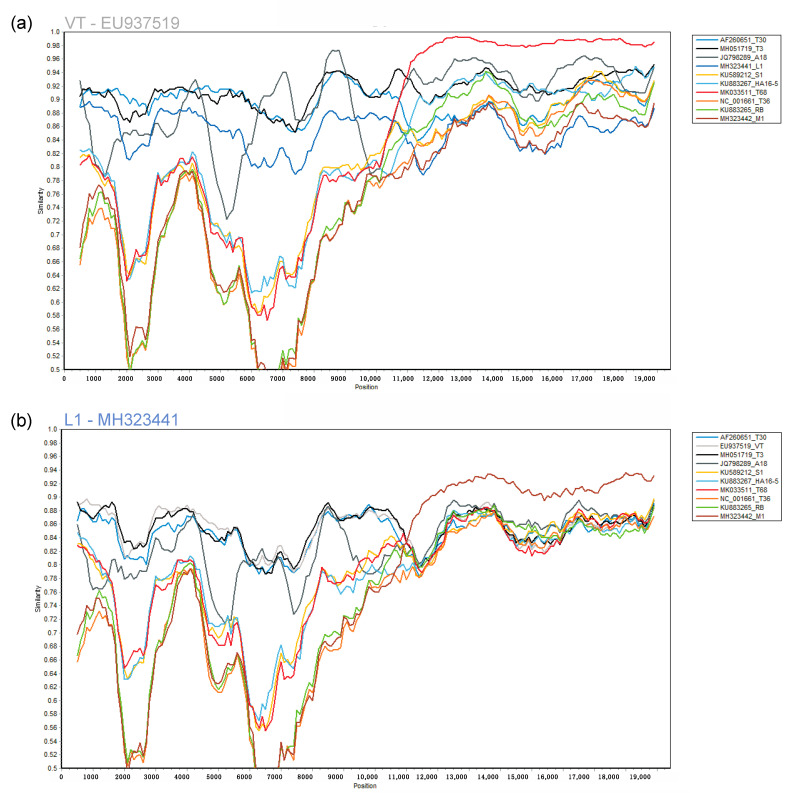
The complete genome of a single citrus tristeza virus (CTV) genotype sequence was compared to a representative sequence of each of the other 10 genotypes at the nucleotide level in a similarity plot. Similarity plots were constructed from a multiple alignment of the eleven genotypes using a window size of 1000 nucleotides (nt) and a step size of 100 nt. (**a**) Genotype sequence VT (EU937519) was selected as the query sequence; (**b**) Genotype sequence L1 (MH323441) was selected as the query sequence.

Neighbor network reconstruction of the complete genomes of CTV revealed eleven genotype groupings (Figure 1). A single accession from each group was selected as the genotype representative sequence. For genotype groups, HA16-5, RB, T3, and T68, a South African sequence was selected that was generated from a plant infected with a single genotype resulting from a single aphid transmission (HA16-5-KU883267, RB-KU883265, T3-MH051719). For genotype groups, T30, T36, VT, and S1, the sequence first reported in the group was selected (T30-AF260651, T36-NC_001661, VT-EU937519, S1-KU589212), and for genotype groups A18, M1, and L1, the available genome was selected (A18-JQ798289, M1-MH323442, L1-MH323441). The eleven selected genotype sequences revealed a maximum diversity based on nucleotide identity of 78.5% between genotype T36 and L1 (Figure 2b). Genotypes VT and T68 are the most similar, sharing a 91.9% nucleotide identity (Figure 2a).

### 3.2. Influence of Citrus Host Reads on Virus Read Mapping

The default CGW read mapping of simulated citrus host reads to the 11 CTV representative genotypes resulted in a maximum of two mapped reads on the different CTV genotypes. The stringent read mapping resulted in no mapped reads.

### 3.3. Single Genotype Infections in Variable Concentrations

**Figure 3:**
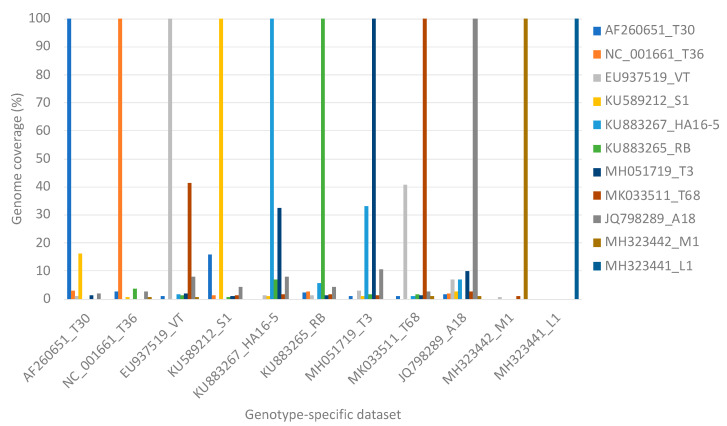
A clustered column chart displaying the genome coverage per representative genotype after read mapping one million read pairs of genotype-specific data on all genotype sequences concurrently.

Genotype specific read mappings against the eleven reference genotypes resulted in more than 99% genome coverage of the target genotype for all data set sizes. Non-target read mappings were observed for all genotypes except for L1, which had only genotype-specific read mappings, utilizing 1000 to 1,000,000 read pairs. The highest number of non-target genome mapping was observed for genotype T68 specific data against genotype VT resulting in a VT genome coverage of a maximum of 41.3% (Figure 3). Genotype VT specific data resulted in a maximum of 40.9% genome coverage of genotype T68 (Figure 3). Genotype T3 specific data resulted in a maximum of 32.5% genome coverage of genotype HA16-5, and genotype HA16-5 specific data resulted in a maximum genome coverage of 33.3% for genotype T3 (Figure 3).

**Figure 4:**
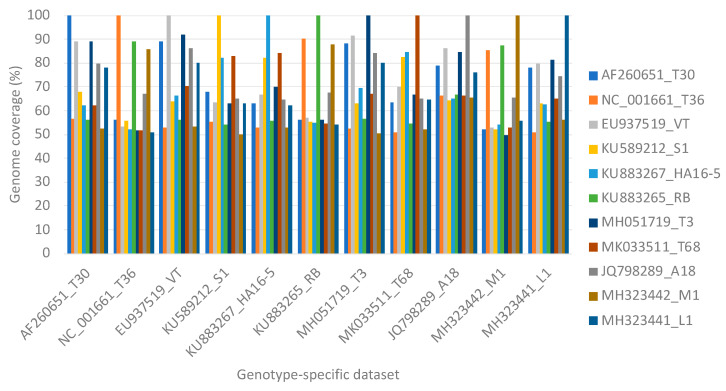
A clustered column chart displaying the genome coverage per representative genotype after read mapping one million read pairs of genotype-specific data on each of the genotype sequences individually.

After performing read mapping on a single genotype reference sequence at a time with each of the genotype-specific data sets, a much higher non-target genome coverage was obtained. The highest non-target genome coverage was obtained for genotype A18 (54.2% with the 1000 read pairs subset of genotype VT) and genotype VT (91.7% with the 1,000,000 read pairs subset of genotype T3) (Figure 4).

### 3.4. Mixed Genotype Infections with Equal Concentrations of Each Genotype

The minimum genome coverage with 1000 read pairs for any of the 11 selected genotype accessions was 99.58% for T30 (AF260651). Genotypes T30, T36, S1, HA16-5, RB, and T3 all reached 100% genotype coverage with 50,000 read pairs. Genotype VT only required 10,000 read pairs to reach 100% genome coverage, while genotype T68 reached 100% genotype coverage with 500,000 read pairs. Genotypes A18, M1, and L1 all reached 100% genome coverage with 100,000 read pairs.

**Figure 5:**
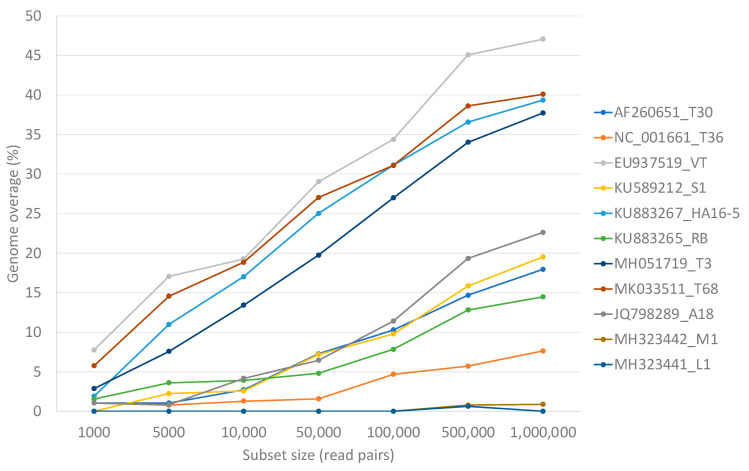
A line graph displaying the non-target genome coverage per representative genotype after read mapping equal numbers of read pairs of each of the other 10 genotype-specific data sets. The number of read pairs per genotype-specific data set was increased to illustrate the influence of sequencing depth on the expected non-target genome coverage.

Mixed genotype infections of equal concentration of 10 of the 11 genotypes resulted in higher numbers of non-target read mappings, and as a result, higher genome coverage for genotypes VT (47.1%), T68 (40.1%), HA16-5 (39.3%), and T3 (37.7%) irrespective of data set size compared to the single variant infection mappings (Figure 5).

### 3.5. Mixed Genotype Infections with Varied Concentrations

The mixing of genotype-specific data sets with a read pair count of either 1000 or 10,000,000 in all the different combinations that the eleven genotypes can occur in a natural infection resulted in the detection of the correct genotypes (genome coverage above 90%) when the genotype-specific data were added to the data pool. None of the genotypes obtained non-target read mappings to the degree that a genome coverage higher than 90% was reached when the genotype-specific data were not added to the data pool. A maximum genome coverage for all genotypes of 99.6% to 100% was obtained when the genotype-specific data were present.

The highest number of non-target read mappings was observed for genotype VT (47.7% genome coverage), followed by genotype T68 (42.1% genome coverage), genotype HA16-5 (40.2% genome coverage), and genotype T3 (37.9% genome coverage). Forty-five different combinations of data sets resulted in a non-target VT genome coverage of 47.7%, of which all data set combinations had the presence of one million read pairs of genotype RB, T68, and A18 (Table 2). The 42.1% T68 non-target genome coverage was obtained with 18 different combinations, which all had one million read pairs of VT, HA16-5, RB, and A18 and 1000 read pairs of T30, T36, and S1. The 40.2% genome coverage obtained by non-target read mappings on HA16-5 was observed with nine combinations, all including one million read pairs of genotypes S1, RB, T3, and A18. The non-target read mappings on T3 that resulted in 37.9% genome coverage in 54 data set combinations all contained one million read pairs of HA16-5, T68, and A18. A 25.3% non-target genome coverage was obtained for genotype A18 with nine different combinations, all containing one million read pairs of genotype T36, VT, S1, HA16-5, RB, T3, and T68.

The results of each of the read mappings performed with the different combinations of data sets are available at https://rbester.shinyapps.io/CTV_mapping_11Dec/ or in Supplementary File S2.

### 3.6. CTV Genotyping of Citrus Plants

The genotype determination RT-PCRs confirmed the CTV genotype status of the citrus samples and confirmed the presence of CTV genotypes RB, VT, T3, T30, and S1 in sample 1, CTV genotype T68 in sample 2, and CTV genotypes RB, VT, T3, T30, and S1 in sample 3. No CTV was detected in sample 4 (healthy control).

The HTS resulted in 24,162,530, 18,215,074, 18,249,385, and 26,952,955 paired reads for the four citrus samples, respectively. After quality trimming, 23,300,914, 15,943,494, 14,497,892, and 25,865,893 read pairs were retained for read mapping for the four samples.

The Blastn results of the de novo assembled contigs of both SPAdes and CGW indicated the presence of CTV, citrus virus A (CiVA), citrus tatter leaf virus (CTLV), hop stunt viroid (HSVd), citrus dwarfing viroid (CDVd), and citrus exocortis viroid (CEVd) in samples 1 and 3. Sample 2 only contained CTV, and no virus contigs were assembled from sample 4 (healthy control).

The read mapping on the eleven selected CTV genotypes revealed the presence of CTV genotypes RB, VT, and T3 in sample 1, CTV genotypes T68 in sample 2, and CTV genotypes RB, VT, and T3 in sample 3 using a genome coverage above 90% as the cut-off for the presence of a genotype (Table 3). Only eight reads of sample 4 (healthy control) mapped to CTV.

To investigate the lower number of read mappings on the expected genotypes T30 and S1, consensus sequences for each of these genotypes were generated from the read data with stringent mapping criteria against accession KC517489 (T30) and KU589212 (S1). The T30 accession was selected based on more reads mapping to KC517489 after the concurrent read mapping of the sample-specific data against all the T30 complete genome sequences available in GenBank. The nt identity between the 10 T30 complete genome sequences available ranged from 98.8% to 100%. The T30 consensus sequence was 95.8%, similar to accession KC517489 for samples 1 and 3, and the S1 consensus sequence was 95.7% and 96.0%, similar to accession KU589212 for samples 1 and 3, respectively. Consensus sequences for each of the other genotypes with a genome coverage above 50% and below 90% (Table 3) were also generated from the read data with stringent mapping criteria against the respective references (HA16-5, T68, and A18).

The read mappings were repeated against the new reference list with the T30, S1, HA16-5, T68, and A18 representative sequences replaced with the consensus sequences. The presence of the consensus sequences did not have an impact on the coverage percentage of the RB, VT, and T3 genotypes for both samples 1 and 3. However, for genotype T30, an increase from 70.5% to 94.2% and 62.3% to 93.2% was observed for samples 1 and 3, respectively (Table 3). For the S1 genotype, a similar increase from 81.5% to 94.6% and 77.1% to 90.5% was observed for samples 1 and 3, respectively (Table 3). The coverage percentage of genotypes HA16-5 and T68 in both samples 1 and 3 did not increase to above 90%; however, the genome coverage of genotype A18 increased from 68.3% to 92.3% in sample 1 and from 58.5% to 88.7% in sample 3 (Table 3).

Read mapping of the data from sample 1 to only the non-target genotype T68 resulted in a genome coverage of 87.3%.

## 4. Discussion

In this study, the use of HTS to differentiate among CTV genotypes was investigated. The level of non-target read mapping was evaluated through the use of simulated and real mixed genotype infections to establish thresholds for genotype-specific genome coverage. By calculating the number of genome bases that are covered at least once, the percentage genome coverage can be determined through read mapping onto a reference genome and serve as a method of virus detection. The confidence in a positive virus/genotype identification is directly correlated with a higher percentage genome coverage. Variation in the number of reads associated with different genomic regions can result in uneven coverage of a viral genome, and as a result, sequencing depth will influence the genome coverage that can be obtained.

A previous study identified at least seven separate CTV genotype groups (RB, T3, T68, T30, VT, HA16-5, and T36) and the single accession JQ798289.1 (A18) [44]. In the present study, a representative of each of these genotype groups and JQ798289.1 (A18), KU589212.1 (S1), MH323441.1 (L1), and MH323442.1 (M1) was selected to represent the diversity within the CTV species (Figure 1).

Before the genotype-specific simulated data were created, the effect of host background reads in the data set was evaluated through performing read mappings on the eleven selected CTV sequences using *Citrus sinensis* host genome-specific reads. Both relaxed and stringent read mapping criteria resulted in no significant number of reads mapping to any of the CTV genotypes, and as a result, the simulated genotype-specific data were created without a host background.

The 11 selected CTV genomes were evaluated for similarity to identify genomic regions that could result in potential non-target read mapping if the genotypes occurred in mixed infections. The Simplot revealed significant nucleotide similarity between genotypes T68 and VT for the 3′ half of the CTV genome (Figure 2a) and based on pairwise comparisons, these two genomes were also the most similar.

This similarity was also evident in the high level of non-target read mappings obtained when genotype T68 specific data were mapped against genotype VT and inversely. More than 40% genome coverage was obtained with these non-target read mappings. The same principle was illustrated in simulated mixed infections in equal concentrations of 10 of the 11 genotypes, where both VT and T68 had a non-target genome coverage above 40% (Figure 3). With mixed infections with varied concentrations and different numbers of genotypes, the highest number of non-target read mappings was also observed for genotype VT (47% read mappings). More than 34% of the non-target genome coverage could be attributed to genotype T68 (Table 2). The lowest number of non-target read mappings against all genotypes was observed for genotype L1 with a genome coverage ranging from 0% for a single infection to 0.6% for a mixed genotype infection when all the other 10 genotype-specific data sets were present (Figure 5). The low nucleotide identity of the representative sequence of genotype L1 to the other genotypes supports this finding (Figure 2b).

The present study also highlighted the effect of using multiple sequences compared to a single sequence when selecting the reference for read mapping. When selecting only a single genotype as a reference for read mapping for any of the genotype-specific data, the non-target read mapping increased significantly to more than 90% (Figure 4). With only 1000 read pairs of the genotype VT specific data set, more than 50% genome coverage was obtained for genotype A18. One million read pairs of genotype T3 resulted in a VT genome coverage of 91%. This high non-target genome coverage can most likely be attributed to the lack of competition for potential mapping positions. The selection of a single CTV genotype sequence for read mapping will adequately identify the presence of CTV in a sample. However, no read mapping below 92% can be used to identify the presence of a specific genotype with confidence.

Single infections simulated with genotype-specific data sets showed that when each of these data sets was concurrently mapped to the eleven reference genotypes, a genome coverage of more than 99% was obtained for each target genotype for all data set sizes (Figure 3). Simulated mixed infections of equal concentrations of 10 of the 11 genotypes showed the same where the minimum genome coverage with 1000 read pairs of the target genotype was higher than 99%. Each of the genotypes obtained a 100% genome coverage if the data pool included 500,000 read pairs of the targeted genotype data set. The simulated mixed infections with varied concentrations and different numbers of genotypes resulted in the detection of the correct genotype with high confidence (genome coverage above 99%) when the genotype-specific data were added to the data pool.

The read mappings using the simulated data revealed that accurate genotype detection was possible in single and mixed infections in all combinations tested. All combinations showed a genome coverage of above 99% when the genotype was present in the data. However, mixed infections resulted in non-target read mappings causing genome coverage of up to 48% for non-target genotypes.

The genome coverage thresholds obtained using the simulated data was evaluated using real HTS data sets generated using well-characterized citrus plants infected with a pre-determined set of CTV genotypes. Read mapping of the data set containing only one CTV genotype confirmed the presence of the T68 genotype in sample 2 (100% genome coverage), with the highest non-target genome coverage obtained for the RB genotype (38%). The other two samples were both expected to contain five genotypes, of which three (RB, T3, and VT) were easily detected based on a genome coverage above 98% after read mapping. The other two genotypes obtained a genome coverage of 70.5% (T30) and 81.5% (S1) for sample 1 and 62.3% (T30) and 77.1% (S1) for sample 3. These genome coverage percentages were higher than the expected non-target genome coverage observed in the simulation experiment. Since the RT-PCR assays also confirmed the presence of these two genotypes, the presence of genotype variants was suspected. In an attempt to characterize these variants, a consensus genome of each of these variants was extracted and compared to the genotype representative sequences previously selected. Limited sequence information is available for the S1 genotype group, and the two consensus sequences were compared to the S1 accession used in this study and found to be 96% similar on nucleotide level. The two T30 variant consensus sequences were also only 96% similar to T30 (KC517489), which currently falls beyond the nt identity range (98.8%) of the 10 complete genome sequence available for the T30 genotype group. However, the complex mixed infection in these two samples makes the generation of a true representative genome per genotype from HTS data difficult, and, therefore, the generation of these consensus genomes was only an attempt to investigate the T30 diversity in these samples. An alternative explanation for the lower genome coverage can also be that these variants represent recombinant sequences. The presence of recombinant virus genomes can complicate the identification of genotypes, and the number of genotypes identified can be an overestimation. Since the RT-PCR only targets a small section of the CTV genome, the positive RT-PCR result only suggests that the target region of T30 or S1 was present in the plant RNA. Recombination has been shown to occur and play an important role in CTV evolution [32,57,58,59,60,61]. The replacement of the representative T30, S1, HA16-5, T68, and A18 genotype sequences with the consensus sequences of each sample increased the genome coverage to above 88% for genotypes T30, S1, and A18 without impacting the genome coverage of the other genotypes present in the samples (Table 3). This would suggest that these samples contained variants of these genotypes and that the selected references did not adequately represent these variants. The similarity between the genotypes present in samples 1 and 3 was the result of tree 1 being the CTV source with which tree 3 was inoculated. The presence of the five genotypes confirmed with RT-PCR was detected in both samples 1 and 3 using the BWA pipeline and a genome coverage threshold of 90%. Based on these criteria, the presence of an A18 genotype variant was also suspected in samples 1 and 3.

To test the effect of using only one genotype at a time as a reference for read mapping, the data from sample 1 were mapped to the non-target T68 genotype, and an 87% genome coverage was obtained. This illustrated that to identify genotypes in mixed infections, a comprehensive approach with regard to CTV reference sequences is needed and that a single reference sequence can result in false-positive results depending on the genome coverage threshold selected. The simulation data using one million read pairs of a specific genotype resulted in a maximum non-target genome coverage of up to 92%. Together with the real data scenario, the threshold for using single reference sequences will have to be above 92% based on the bioinformatic pipeline and the sequencing depth presented here.

The more comprehensive approach will be to include multiple CTV references representing different genotypes in the read mapping reference list. The analyses of both the simulated and real data sets showed that a genome coverage below 50% is indicative of non-targets or false positive genotype results. A previous simulated study using a different bioinformatic pipeline and fewer CTV reference sequences also showed non-target read mappings resulting in a maximum of 90% genome coverage for single and mixed CTV infections [54]. This previous study also showed that a 95% genome coverage is indicative of the presence of a genotype and that this 95% was attainable when 10,000 single end simulated reads mapped to a specific genotype [54]. In the present study, these findings were confirmed, and it was demonstrated that greater sequencing depth (simulated with different data set sizes) impacted the level of non-target mappings observed (Figure 5); however, a 99% genome coverage was attained for all expected genotypes with only 1000 read pairs mapped.

The diversity within the CTV species and the continuous identification of new genotypes or variants complicates the development of a one size fits all CTV genotyping tool. However, the results presented here provide a valid pipeline to identify the known genotypes to date with high confidence. The presence of novel or recombinant sequences will most likely present with a genome coverage above the 50% threshold as in the real data examples. This threshold will distinguish false positives read mappings but enable the identification of potential novel genotypes.

## 5. Conclusions

In this study, an HTS bioinformatic pipeline was established to identify CTV genotypes in single or mixed infections. This bioinformatic pipeline utilized read mapping against a sequence list representing different CTV genotypes. Simulated and real HTS data sets were used to identify genome coverage thresholds to categorize false positive read mappings and true genotype-specific genome coverage. Using this read mapping pipeline, genome coverage below 50% was regarded as the result of non-target read mappings. A genome coverage above 90% was indicative of the presence of a specific genotype, and genome coverage between 50–90% suggested the presence of genotype variants not represented in the read mapping reference list. The abovementioned thresholds were all dependent on at least 1000 read pairs of CTV mapping to a genotype sequence. These thresholds will also be influenced by genotype concentration and sequencing depth of the library, which was not necessarily investigated to the extremes in this study.

The use of the real HTS data and the identification of the potential genotype variants highlight the need for a continuous investigation into the diversity of CTV and to develop accurate reference databases to define genotype group boundaries. This will enable further biological characterization and population studies and potentially improve the understanding of cross-protection.

This study evaluated the specificity of a CTV genotyping bioinformatic pipeline and proposed the use of HTS to identify CTV genotypes in citrus.

## Figures and Tables

**Table 1:** Citrus tristeza virus (CTV) species and genotype-specific primer sequences used in a two-step RT-PCR.

Primer Name	Polarity	Primer Sequences (5′ to 3′)	Ta (°C) ^1^	Amplicon Size (bp)	Reference
CTV generic	Sense	TCTGATTGAAGTGGACGGAATAAG	62	157	[33] ^2^
	Anti-sense	GCTTAGACCAACGAGAGGATA			
RB: group1 ^3^	Sense	AGTGGTGGAGATTACGTTG	60	628	[33]
	Anti-sense	TACACGCGACAAATCGAG			
RB: group 2 ^4^	Sense	CGGAAGGGACTACGTGGT	60	658	[33]
	Anti-sense	CGTTTGCACGGGTTCAATG			
T36	Sense	GGTGTAAGGAAGCGTGTGTCGCATTTA	66	537	[33]
	Anti-sense	ACCTGCACCGTCTAACAACATCATCG			
HA16-5	Sense	CGACAAGTGCATTACGTCTCAG	56	176	[33]
	Anti-sense	GTAAGTATCTAAAACCAGGAG			
T68 (B165)	Sense	GTTAAGAAGGATCACCATCTTGACGTTGA	64	510	[45] ^5^
	Anti-sense	AAAATGCACTGTAACAAGACCCGACTC			
T3	Sense	GTTATCACGCCTAAAGTTTGGTACCACT	60	409	[45]
	Anti-sense	CATGACATCGAAGATAGCCGAAGC			
VT	Sense	TTTGAAAATGGTGATGATTTCGCCGTCA	60	302	[45]
	Anti-sense	GACACCGGAACTGCYTGAACAGAGT			
T30	Sense	TGTTGCGAAACTAGTTGACCCTACTG	60	206	[45]
	Anti-sense	TAGTGGGCAGAGTGCCAAAAGAGAT			
S1	Sense	CGACGAGTATATGAAGGACAAC	52	715	Citrus Research International (CRI)
	Anti-sense	GAAAACCCGTAGCTGTCTAATGC			

^1^ Annealing temperature. ^2^ Cook, G.; van Vuuren, S.P.; Breytenbach, J.H.J.; Burger, J.T.; Maree, H.J. Expanded Strain-Specific RT-PCR Assay for Differential Detection of Currently Known Citrus Tristeza Virus Strains: a Useful Screening Tool. *Journal of Phytopathology* 2016, 164, 847–851, doi:10.1111/jph.12454. ^3^ RB group 1 included genotypes NZRB-TH28, NZRB-M12, NZRB-G90, and HA18-9. ^4^ RB group 2 included genotypes NZRB-TH30, NZRB-M17, and Taiwan-Pum/SP/T1. ^5^ Roy, A.; Ananthakrishnan, G.; Hartung, J.S.; Brlansky, R.H. Development and Application of a Multiplex Reverse-Transcription Polymerase Chain Reaction Assay for Screening a Global Collection of Citrus tristeza virus Isolates. *Phytopathology*, 2010, 100, 1077–1088, doi:10.1094/PHYTO-04-10-0102.

**Table 2:** Non-target genome coverage (%) of genotype VT (EU937519) after read mapping all the other ten genotype specific data sets and then subtracting each of the genotype-specific data sets to identify the major contributor to the VT non-target read mapping. A “0” represents no data were added to the pool of reads used for the specific read mapping event, and a “7” represents that the 7th largest data set (one million read pairs) was added to the pool of reads used for the specific read mapping event.

Genotype-Specific Data Set											Genome Coverage (%)
AF260651 T30	NC_001661 T36	EU937519 VT	KU589212 S1	KU883267 HA16-5	KU883265 RB	MH051719 T3	MK033511 T68	JQ798289 A18	MH323442 M1	MH323441 L1	
7	7	0	7	7	7	7	7	7	7	7	47.1
0	7	0	7	7	7	7	7	7	7	7	47.7
7	0	0	7	7	7	7	7	7	7	7	47.7
7	7	0	0	7	7	7	7	7	7	7	47.7
7	7	0	7	0	7	7	7	7	7	7	47.7
7	7	0	7	7	0	7	7	7	7	7	47.6
7	7	0	7	7	7	0	7	7	7	7	47.7
7	7	0	7	7	7	7	0	7	7	7	12.8
7	7	0	7	7	7	7	7	0	7	7	40.9
7	7	0	7	7	7	7	7	7	0	7	47.1
7	7	0	7	7	7	7	7	7	7	0	47.1

**Table 3:** Genome coverage (%) obtained per genotype after read mapping the real HTS data of the three samples to reference list 1 (one representative genome of each genotype) and reference list 2 (one representative genome of each genotype with the T30, S1, HA16-5, T68 and A18 genomes replaced with read mapping consensus sequences).

Genotype (Reference List 1)	Genome Coverage (%)	Genotype (Reference List 2)	Genome Coverage (%)
Sample 1			
AF260651_T30	70.5	KC517489.1_T30_consensus	93.8
NC_001661_T36	40.9	NC_001661_T36	38.2
EU937519_VT	99.9	EU937519_VT	98.9
KU589212_S1	81.5	KU589212_S1_consensus	93.9
KU883267_HA16-5	59.4	KU883267_HA16-5_consensus	63.2
KU883265_RB	100.0	KU883265_RB	100.0
MH051719_T3	98.8	MH051719_T3	98.4
MK033511_T68	59.4	MK033511_T68_consensus	69.0
JQ798289_A18	68.3	JQ798289_A18_consensus	92.3
MH323442_M1	24.7	MH323442_M1	24.7
MH323441_L1	17.1	MH323441_L1	15.3
Sample 2			
AF260651_T30	6.5		
NC_001661_T36	2.8		
EU937519_VT	43.8		
KU589212_S1	13.4		
KU883267_HA16-5	12.6		
KU883265_RB	39.0		
MH051719_T3	21.6		
MK033511_T68	100.0		
JQ798289_A18	16.3		
MH323442_M1	2.9		
MH323441_L1	2.1		
Sample 3			
AF260651_T30	62.3	KC517489.1_T30_consensus	92.8
NC_001661_T36	33.9	NC_001661_T36	34.1
EU937519_VT	99.9	EU937519_VT	95.3
KU589212_S1	77.1	KU589212_S1_consensus	90.6
KU883267_HA16-5	55.5	KU883267_HA16-5_consensus	59.7
KU883265_RB	100.0	KU883265_RB	100.0
MH051719_T3	98.2	MH051719_T3	96.8
MK033511_T68	60.9	MK033511_T68_consensus	67.5
JQ798289_A18	58.5	JQ798289_A18_consensus	88.7
MH323442_M1	19.0	MH323442_M1	18.7
MH323441_L1	11.0	MH323441_L1	7.7

## Data Availability

The data presented in this study are available in Supplementary File S2: Genome_coverage_all_combinations.txt or at https://rbester.shinyapps.io/CTV_mapping_11Dec/.

## Supplementary Materials

The following are available online at https://www.mdpi.com/1999-4915/13/2/168/s1, Supplementary File S1: Read_mapping_pipeline.sh; Supplementary File S2: Genome_coverage_all_combinations.txt; Supplementary File S3: S3_Reference_list2_sample1.fasta; Supplementary File S4: S4_Reference_list2_sample3.fasta; Supplementary File S5: S5_Sample1_Mapping_results3.txt; Supplementary File S6: S6_Sample2_Mapping_results3.txt; Supplementary File S7: S7_Sample3_Mapping_results3.txt.
